# An Update on the Controversies in Anemia Management in Chronic Kidney Disease: Lessons Learned and Lost

**DOI:** 10.1155/2011/623673

**Published:** 2011-04-10

**Authors:** Geoffrey Teehan, Robert L. Benz

**Affiliations:** ^1^Department of Medicine, Division of Nephrology, Lankenau Medical Center, Suite 130 MOBW, 100 Lancaster Avenue, Wynnewood, PA 19096, USA; ^2^Lankenau Institute for Medical Research, 100 Lancaster Avenue, Wynnewood, PA 19096, USA

## Abstract

*Background*. Erythropoietin deficiency and anemia occur in Chronic Kidney Disease (CKD) and may be treated with Erythropoietin Stimulating Agents (ESAs). The optimal hemoglobin, in non-End Stage Renal Disease CKD, is controversial. *Methods*. We review three recent randomized trials in anemia in CKD: CHOIR, CREATE, and TREAT. *Results*. CHOIR (*N* = 1432) was terminated early with more frequent death and cardiovascular outcomes in the higher Hb group (HR 1.34: 95% C.I. 1.03–1.74, *P* = .03). CREATE (*N* = 603) showed no difference in primary cardiovascular endpoints. Stroke was more common in the higher Hb group (HR 1.92; 95% C.I. 1.38–2.68; *P* < .001) in TREAT (*N* = 4038). *Conclusions*. There is no benefit to an Hb outside the 10–12 g/dL range in this population. To avoid transfusions and improve Quality of Life, ESAs should be used cautiously, especially in patients with Diabetes, CKD, risk factors for stroke, and ESA resistance.

## 1. Introduction

Anemia is an expected feature of chronic kidney disease (CKD) once the glomerular filtration rate (GFR) drops below 60 mL/minute. The World Health Organization (WHO) defines anemia as a hemoglobin (Hb) below 13 g/dL for adult males and postmenopausal women, and below 12 g/dL for premenopausal women [1]. The anemia of CKD, due primarily to erythropoietin deficiency, is usually normochromic, normocytic, associated with shortened red blood cell survival, and some degree of iron deficiency [2]. Prospective cohort studies suggest a 1% prevalence of anemia for a GFR of 60 mL/minute, rising to 9% below 30 mL/minute, and 33–67% for those with a GFR below 15 mL/minute [2]. 

Recombinant human erythropoietin (rHU EPO) has been available for the treatment of anemia of CKD since the 1980s and supplanted the use of blood transfusions and adrogenic steroids [3]. Improved quality of life scores was a consistent finding in early studies involving rHU EPO and CKD [4–6]. Nevertheless, even early literature revealed accelerated hypertension, failure of vascular accesses, and occasionally hyperkalemia [7]. Later, as the link between cardiovascular disease (CVD) and CKD became more evident, it became clear that anemia was an independent risk factor for developing Left Ventricular Hypertrophy and Heart Failure [8]. Lacking the benefits of any randomized, controlled, clinical trials, use of Erythropoietin Stimulating Agents (ESAs) became widespread, and the optimal hemoglobin to limit cardiovascular events was unknown [9, 10].

Restoring the Hb to normal levels in patients with ESRD on hemodialysis may be associated with an improvement in quality of life and regression of LVH but possibly at the expense of serious cardiovascular outcomes, particularly among those in whom anemia is more difficult to correct [10].  Table 1 lists various recommendations for target Hb. Until 2006, the most consistent finding seemed to be that an Hb level between 11–13 g/dL improved quality of life, without increasing CVD risk [11–16]. Still, as several trials have explored higher Hb targets, outcomes such as more rapid progression of cancers and increased risk of death and serious cardiovascular events have prompted a black box warning on all ESAs [17–19]. 

Possibly lost in all of this is the wealth of data accumulated in the 1980s and 1990s showing improved quality of life scores and increased vitality. Among ESRD patients receiving ESAs, Benz et al. showed statistically significant improvements in Maintenance of Wakefullness Testing (MWT), reductions in Arousing Periodic Limb Movements (PLMS), and interestingly, the hematocrit in these patients was normalized (mean hematocrit 42.3%) [4]. Revicki et al. in 1995 demonstrated significant improvements in assessments of energy (*P* < .05), physical function (*P* < .05), home management (*P* < .05), social activity (*P* < .05), and cognitive function (*P* < .05) among CKD patients assigned to receive ESA versus conservative therapy without ESAs. In this trial, 79% of ESA treated patients achieved a Hematocrit level over 36% [6]. In 1991, 117 patients with anemia related to chronic renal failure not yet requiring dialysis were randomized to receive erythropoietin to correct anemia (hematocrit of 40% for males, 35% for females) versus placebo. Energy levels and work capacity improved significantly in the group with corrected anemia compared with the group with uncorrected anemia [5]. Painter demonstrated improvements in exercise training with rHU EPO in patients with ESRD [20]. Finally, Moia et al. showed a reduction in bleeding time by increasing the Hb through the use of rHU EPO [21].

This review will focus on the three most recent clinical trials (CHOIR, CREATE, and TREAT), each of which assesses the optimal Hb in patients with CKD stages III-V and the associated cardiovascular outcomes [10, 22, 23].  Table 2 outlines the characteristics of each trial.

## 2. CREATE

The Cardiovascular Risk Reduction by Early Anemia Treatment with Epoetin Beta trial (CREATE), published in 2006, was a randomized, controlled, clinical trial enrolling 603 patients to study the cardiovascular benefit of Epoetin Beta in anemic patients (Hb level 11–12.5 g/dL) with stages III-IV CKD (GFR 15–35 mL/minute/1.73 m^2^). Patients were randomly assigned to a target Hb in the normal range (13–15 g/dL, *n* = 301) versus a subnormal level (10.5–11.5 g/dL, *n* = 302). The primary endpoint was a composite of 8 cardiovascular events which included time to a first cardiovascular event, sudden death, myocardial infarction, acute heart failure, stroke, transient ischemic attack, angina pectoris, or cardiac arrhythmia resulting in hospitalization, and complication of peripheral vascular disease. 

Secondary endpoints included death from any cause, death from cardiovascular causes, and hospitalization for any cause among others. Patients who were expected to require renal replacement therapy within six months, had advanced cardiovascular disease, were recently transfused, or had non-renal causes of anemia were excluded.

The study was powered to detect an annual reduced incidence of primary endpoint of 15%. Initial demographic data showed that the groups were largely similar with very few exceptions. The vast majority of patients in the normal Hb group received Epoietin beta (98%). In the subnormal Hb group, 32% received Epoietin Beta in year one, 52% in year two, and 76% at the end of the study. At the end of study, the Hb levels between the two groups differed by 1.5 g/dL. 

Compared to the normal Hb group, the subnormal Hb group did not experience significantly more first cardiovascular events or decline in GFR, but time to initiation of dialysis after 18 months of the trial was significantly shorter among those treated to a normal Hb (*P* = .03). Nevertheless, fully 105 primary cardiovascular events occurred (58 in the higher Hb group versus 47 in the lower Hb group, *P* = NS). General health and physical function were significantly improved relative to the subnormal Hb group (*P* = .003 and *P* < .001, resp.). The investigators concluded that early complete correction of anemia did not reduce the risk of cardiovascular events among anemic patients with stage III-IV CKD.

## 3. CHOIR

The results of the Correction of Hemoglobin in Outcomes and Renal Insufficiency (CHOIR) trial were also reported in 2006. The open-label randomized, controlled, clinical trial enrolling 1432 patients with anemia (all with Hb below 11 g/dL at enrollment, and naïve to ESAs) and CKD III-IV (GFR between 15–50 mL/minute/1.73 m^2^) compared cardiovascular and renal outcomes for two groups randomized to receive Epoetin Alfa to achieve mean Hbs of 11.3 g/dL (*N* = 717) versus 13.5 g/dL (*N* = 715). The primary end point was the time to the composite of death, myocardial infarction, hospitalization for congestive heart failure (with the exclusion of renal replacement therapy), or stroke. Secondary outcomes included the time to renal replacement therapy, hospitalization for either cardiovascular causes or any cause, and quality of life. Patients with uncontrolled hypertension, active gastrointestinal bleeding, iron overload, history of frequent transfusions, refractory iron deficiency anemia, active cancer, angina pectoris, or previous ESA treatment were excluded.

At baseline, patients in both groups were very similar with respect to their demographics with few exceptions. Originally, patients were to be followed for approximately 3 years and the study was powered to show a 25% reduction in the composite event rate in the higher Hb group. All 1432 patients were included in the final analysis in an intention to treat model. Median followup was 16 months. 

After 3 months, a difference in Hb values between the 2 groups was observed. Although the low Hb group was largely able to reach the prespecified target Hb, fewer patients in the high Hb group were able to reach their prespecified target Hb. Not surprisingly, the low Hb group required a significantly lower dose of Epoetin alfa.

By May of 2005, the trial was terminated at the recommendation of the data and safety monitoring board after recording 125 events in the higher Hb group versus 97 events in the lower Hb group (HR, 1.34; 95% confidence interval, 1.03–1.74; *P* = .03). These included deaths, myocardial infarctions, strokes, and hospitalizations for congestive heart failure. They concluded that the likelihood of showing a benefit in the higher Hb group was negligible. Although no individual primary outcome reached statistical significance, there appeared to be a trend among the higher Hb group toward hospitalization for CHF (HR 1.41, *P* = .07) and death (HR 1.48, *P* = .07). Quality of life scores were similar among the 2 groups.

## 4. TREAT

More recently, the Trial to Reduce cardiovascular Events with Aranesp Therapy (TREAT) was conducted to look at patients who are anemic (Hb less than 11 g/dL) and had Diabetic CKD III-IV (GFR 20–60 mL/minute/1.73 m^2^). This randomized, controlled, clinical trial enrolled over 4000 patients and randomly assigned approximately 2000 patients to be treated with Darbepoetin Alfa to achieve an Hb of approximately 13 g/dL, and the remainder of patients received placebo and rescue Darbepoetin Alfa therapy if and when the Hb fell below 9 g/dL. The primary end points included the composite outcomes of death or cardiovascular event and a composite outcome of time to death or end-stage renal disease. Secondary endpoints included time to death, death from cardiovascular causes, rate of GFR decline, and quality of life measures.

Relevant exclusion criteria included those with uncontrolled hypertension, previous cardiovascular events or kidney transplantation, chemotherapy/radiation therapy, cancer, hematologic diseases, pregnancy, and those receiving ESA therapy in the preceding 12 weeks. The demographics of the patients in Darbepoetin Alfa group (*N* = 2012) and the Placebo group (*N* = 2026) were similar in most respects with the exception of a higher percentage of patients in the Placebo group having a history of cardiovascular disease, higher creatinine at baseline, and lower glycated Hb. 

During this trial, the results of CHOIR were revealed to the enrolled patients, but the study was not terminated. The trial was powered to detect 20% risk reduction for the primary endpoint among those targeted to a higher Hb. 

Three months after the initiation of the trial, the median achieved Hb level in the Darbepoetin Alfa group differed significantly from that in the placebo group (12.5 g/dL versus 10.6 g/dL, *P* < .001). At the end of the trial, there were no differences in the primary cardiovascular composite endpoints (31.4% of patients in the treatment arm versus 29.7% of patients in the placebo arm reached a cardiovascular composite endpoint, *P* = .41). However, a significantly higher proportion of patients suffered fatal or nonfatal stroke among those treated with Darbepoetin Alfa (5% versus 2.6%, HR 1.92; 1.38–2.68, *P* < .001), despite no evident difference between blood pressures in each group. The renal composite endpoint was reached by 32.4% of patients in the treatment arm and 30.5% in the placebo arm (*P* = .29). A higher proportion of patients in the placebo group required cardiac revascularization (5.8% versus 4.2%, HR 0.71, 0.54–0.94, *P* = .02). Interestingly, a significantly higher proportion of patients randomized to the placebo arm required transfusions versus those in the treatment arm (24.5% versus 14.8%, HR for Aranesp 0.56; 95% confidence interval, 0.49–0.65; *P* < .001). There was a nonstatistically significant trend towards a higher rate of cancer deaths among those in the treated group (39 versus 25, *P* = .08). 

## 5. Conclusions

These three clinical trials in anemic patients with non-ESRD CKD each showed no clear benefit to normalizing Hb in this population, while in some cases showed harm. At best, these trials do show improvements in quality of life particularly with regard to vitality, physical function, and mental health. The shorter time to dialysis seen in the CREATE trial, a higher composite rate of death, myocardial infarction, and hospitalization for CHF in the early-terminated CHOIR study, and the higher stroke risk in the TREAT trial weigh against these quality of life improvements and suggest that normalizing Hb in this population is not warranted and potentially hazardous. The lack of a difference in cardiovascular outcomes in the placebo-controlled TREAT trial, which enrolled twice as many patients as the other 2 trials combined, is compelling given the paucity of placebo-controlled trials in this population. 

The mean Hb among placebo-treated patients in the TREAT trial was 10.6 g/dL and was 11.3 g/dL and 11.6 g/dL in the lower Hb groups in the CHOIR and CREATE trials, respectively. Still, 46 percent of patients assigned to the placebo arm in TREAT received at least one dose of Darbepoetin alfa. Fully 76% of patients in the lower Hb group in CREATE received Epoetin beta. Among those in the CHOIR, study the mean dose of Epoetin Alfa for the lower Hb group was approximately 6000 units per week. Nevertheless, even among the more conservatively treated patients, the incidence of serious cardiovascular and renal outcomes was not negligible. In both CHOIR and CREATE, approximately 15% of those targeted to a lower Hb experienced a composite endpoint (CHOIR) or a cardiovascular endpoint (CREATE). Death or a cardiovascular endpoint occurred in nearly 30% of patients assigned to the placebo group in TREAT. 

The high prevalence of cardiovascular disease among CKD patients is well known, and this burden of CVD increases with the severity of the CKD [18]. While it is reasonable to surmise that pharmacologically correcting anemia, in lieu of transfusions, to some extent, is beneficial to patients with anemia and CKD, by virtue of reducing well-known transmission of viruses, these trials offer no convincing data to establish a cutoff lower (or upper) limit for anemia management. Eckhardt et al. reviewed echocardiographic data from CREATE and concluded that complete correction of anemia exacerbated the prognosis of eccentric LVH [24]. Weiner et al., however, concluded that amongst patients with CKD in the Atherosclerotic Risk in Communities Study, Cardiovascular Health Study, Framingham Heart Study, and Framingham Offspring Study, LVH and anemia conferred a higher risk of composite cardiovascular outcomes (HR for LVH 1.67 95% CI 1.34–2.07 and HR for Anemia 1.51 95% CI 1.27–1.81). Weiner further showed that the combination of anemia and LVH among patients with CKD conferred greater than four times the risk of these outcomes versus those without either comorbidity [25]. Besarab's data suggests that those requiring progressively higher ESA doses to achieve a target Hb may have higher incidence of death [26].

Even though anemia correction may have a salutary effect on LVH regression and reduction in cardiovascular outcomes [27], this must be weighed against the disappointing findings in TREAT, CHOIR, and CREATE. While in 2009 the K-DOQI Guidelines suggest that the target Hb “generally be in the range of 11-12 g/dL,” in the absence of compelling data to the contrary, and in the spirit of doing no harm, it may be more reasonable to target the Hb as specified by the package inserts which aim for 10–12 g/dL. The highest Hb target among those targeted to a lower Hb in the three trials reviewed was 11.6 in the CREATE trial. Substantial dosage reductions should occur as the Hb approaches 12 g/dL. Furthermore, particular attention should be paid to those who respond slowly and require higher doses of ESAs, and to those with Diabetes and CKD and risk factors for stroke. 

Nevertheless, it would behoove the practitioner to recall the Quality of Life benefits conferred by ESAs in the studies in the 1990s and avoid the knee-jerk response of underutilization of these drugs. One more recent trial, only in abstract form at this point, CAPRIT (The Complete Correction of Post-Transplant Anemia reduces the Rate of Progression of Chronic Allograft Nephropathy) may demonstrate a benefit to a higher hemoglobin. This randomized trial of 128 patients (at least one year after renal transplant) with CKD III-IV (GFR 20–50 mL/min) sought to determine if a higher Hb would be associated with a slower progression of Chronic Allograft Nephropathy. Patients were randomized to a target Hb of 13–15 g/dL (Group A) versus 10.5–11.5 g/dL (Group B). At the end of the study, the Hb in Group A versus B was 12.9 versus 11.3 g/dL (*P* < .001), and after two years 4 (Group A) versus 10 patients (Group B) reached ESRD (*P* < .01). At one year, the respective GFRs were 35.9 ± 17.2 mL/min (Group A) versus 30.8 ± 12.1 mL/min (Group B) (*P* < .025). The incidence of cardiovascular events did not differ between groups [28]. 

In conclusion, ESA use reduces the need for transfusions, improves quality of life and exercise tolerance, can regress LVH, and may be of benefit in retarding Chronic Allograft Nephropathy. ESA use, however, is also associated with poorer cardiovascular outcomes when used to target hemoglobins higher than the prespecified package inserts and possibly when used excessively in the setting of refactory anemia [3, 4, 7, 8, 12, 14, 17, 19]. The data strongly suggest a target Hb in the CKD population no higher than 10–12 g/dL, careful attention to underlying risks for cardiovascular disease, and thorough evaluation of correctable causes of anemia to prevent “overuse” of ESAs.

## Figures and Tables

**Table 1:** Target hemoglobin in CKD/ESRD.

Society	Year	Target Hb (g/dL)
NKF-DOQI [16]	2007	11-12
Canadian Society of Nephrology [11]	1999	11-12
Japanese Society for Dialysis Therapy [13, 14]	2004	10-11, 11-12*
ERA-EDTA [12, 15]	2004	≥11**

*In active younger patients.

**With no upper limit.

**Table 2:** Study characteristics.

Study	*N* (pts)	HB target (g/dL)	ESA	GFR range (mL/min/1.73 m^2^)	Primary endpoint	*P* value for primary endpoints
CHOIR (2006) [10]	603	13.5 versus 11.3	Epoetin Alfa	15–50	Death, MI, CHF, CVA	0.03 for composite favoring lower Hb
CREATE (2006) [22]	1432	13–15 versus 10.5–11.5	Epoetin Beta	15–35	Composite of 8 CV events, CKD progression	NS for CV events. 0.03 for ESRD favoring lower Hb group
TREAT (2009) [23]	4038	13 versus 9	Darbepoetin Alfa	20–60	Death, CV Event, ESRD	NS

## References

[1] World Health Organization (1968). Nutritional Anaemias: Report of a WHO scientific group.

[2] Astor BC, Muntner P, Levin A, Eustace JA, Coresh J (2002). Association of kidney function with anemia. The Third National Health and Nutrition Examination Survey (1988–1994). *Archives of Internal Medicine*.

[3] Obrador GT, Roberts T, St. Peter W.L. WL, Frazier E, Pereira BJG, Collins AJ (2001). Trends in anemia at initiation of dialysis in the United States. *Kidney International*.

[4] Benz RL, Pressman MR, Hovick ET, Peterson DD (1999). A preliminary study of the effects of correction of anemia with recombinant human erythropoietin therapy on sleep, sleep disorders, and daytime sleepiness in hemodialysis patients (the SLEEPO study). *American Journal of Kidney Diseases*.

[5] Teehan BP, Krantz S, Stone WA (1991). Double-blind, placebo-controlled study of the therapeutic use of recombinant human erythropoietin for anemia associated with chronic renal failure in predialysis patients. *American Journal of Kidney Diseases*.

[6] Revicki DA, Brown RE, Feeny DH (1995). Health-related quality of life associated with recombinant human erythropoietin therapy for predialysis chronic renal disease patients. *American Journal of Kidney Diseases*.

[7] Winearls CG (1995). Historical review on the use of recombinant human erythropoietin in chronic renal failure. *Nephrology Dialysis Transplantation*.

[8] McClellan WM, Flanders WD, Langston RD, Jurkovitz C, Presley R (2002). Anemia and renal insufficiency are independent risk factors for death among patients with congestive heart failure admitted to community hospitals: a population-based study. *Journal of the American Society of Nephrology*.

[9] Phrommintikul A, Haas SJ, Elsik M, Krum H (2007). Mortality and target haemoglobin concentrations in anaemic patients with chronic kidney disease treated with erythropoietin: a meta-analysis. *Lancet*.

[10] Singh AK, Szczech L, Tang KL (2006). Correction of anemia with epoetin alfa in chronic kidney disease. *New England Journal of Medicine*.

[11] Barrett BJ, Fenton SS, Ferguson B (1999). Clinical practice guidelines for the management of anemia coexistent with chronic renal failure. Canadian Society of Nephrology. *Journal of the American Society of Nephrology*.

[12] Cameron JS (1999). European best practice guidelines for the management of anaemia in patients with chronic renal failure. *Nephrology Dialysis Transplantation*.

[13] Gejyo F, Saito A, Akizawa T (2004). 2004 Japanese Society for Dialysis Therapy guidelines for renal anemia in chronic hemodialysis patients. *Therapeutic Apheresis and Dialysis*.

[14] Hirasawa Y, Itami N, Mizuno T (2003). Maintenance hamatocrit levels and mortality in hemodialysis patients with renal anemia receiving recombinant human erythropoietin(rHuEPO) treatment(rHuEPO survey). *Journal of Japanese Society for Dialysis Therapy*.

[15] Locatelli F, Nissenson AR, Barrett BJ (2008). Clinical practice guidelines for anemia in chronic kidney disease: problems and solutions. A position statement from Kidney Disease. Improving Global Outcomes (KDIGO). *Kidney International*.

[16] National Kidney Foundation-KDOQI (2007). Clinical practice guidelines and clinical practice recommendations for anemia in chronic kidney disease. *American Journal of Kidney Diseases*.

[17] Bohlius J, Schmidlin K, Brillant C (2009). Recombinant human erythropoiesis-stimulating agents and mortality in patients with cancer: a meta-analysis of randomised trials. *The Lancet*.

[18] FDA FDA black box warning for Aranesp. Silver Spring, MD: food and drug administration. http://www.accessdata.fda.gov/drugsatfda_docs/label/2008/103951s5195PI.pdf.

[19] Leyland-Jones B, Semiglazov V, Pawlicki M (2005). Maintaining normal hemoglobin levels with epoetin alfa in mainly nonanemic patients with metastatic breast cancer receiving first-line chemotherapy: a survival study. *Journal of Clinical Oncology*.

[20] Painter P (1994). The importance of exercise training in rehabilitation of patients with end-stage renal disease. *American Journal of Kidney Diseases*.

[21] Moia M, Vizzotto L, Cattaneo M, Mannucci PM, Casati S, Ponticelli C (1987). Improvement in the haemostatic defect of uraemia after treatment with recombinant human erythropoietin. *Lancet*.

[22] Drüeke TB, Locatelli F, Clyne N (2006). Normalization of hemoglobin level in patients with chronic kidney disease and anemia. *New England Journal of Medicine*.

[23] Pfeffer MA, Burdmann EA, Chen CY (2009). A trial of darbepoetin alfa in type 2 diabetes and chronic kidney disease. *New England Journal of Medicine*.

[24] Eckardt KU, Scherhag A, Macdougall IC (2009). Left ventricular geometry predicts cardiovascular outcomes associated with anemia correction in CKD. *Journal of the American Society of Nephrology*.

[25] Weiner DE, Tighiouart H, Vlagopoulos PT (2005). Effects of anemia and left ventricular hypertrophy on cardiovascular disease in patients with chronic kidney disease. *Journal of the American Society of Nephrology*.

[26] Besarab A, Bolton WK, Browne JK (1998). The effects of normal as compared with low hematocrit values in patients with cardiac disease who are receiving hemodialysis and epoetin. *New England Journal of Medicine*.

[27] Levin A (2002). Anemia and left ventricular hypertrophy in chronic kidney disease populations: a review of the current state of knowledge. *Kidney International, Supplement*.

[28] Oral A (2010). the complete correction of post-transplant anemia reduces the rate
of progression of chronic allograft nephropathy. *American Journal of Transplantation*.

